# Conditional Melanoma Cancer Survival in the United States

**DOI:** 10.3390/cancers8020020

**Published:** 2016-02-02

**Authors:** Ray M. Merrill, Simone Bateman

**Affiliations:** Department of Health Science, Brigham Young University, Provo, UT 84602, USA; Simonebateman001@gmail.com

**Keywords:** cancer, conditional survival, population-based, prognosis, relative survival, SEER

## Abstract

Beyond relative survival, which indicates the likelihood that patients will not die from causes associated with their cancer, conditional relative survival probabilities provide further useful prognostic information to cancer patients, tailored to the time already survived from diagnosis. This study presents conditional relative survival for melanoma patients in the United States, diagnosed during 2000–2008 and followed through 2012. Analyses are based on 62,803 male and 50,261 female cases in population-based cancer registries in the Surveillance, Epidemiology, and End Results Program of the National Cancer Institute. Five-year relative survival estimates are presented for melanoma patients who have already survived one, two, three, four, or five years after the initial diagnosis. Five- and ten-year relative survival decreases with age, stage at diagnosis, and is lower among males, Blacks, and Hispanics. Five-year conditional relative survival improves with each year already survived. The potential for improvement in five-year conditional relative survival is greatest for older age, males, Blacks, Hispanics, and in later staged cases. For local disease, five-year conditional relative survival was significantly lower in ages greater than 65 years and in Blacks. It was significantly higher in females, non-Hispanics, and married individuals. Age had a greater inverse relationship with five-year survival in later staged disease. A similar result occurred for females and married individuals. In contrast, non-Hispanics had better five-year survival if diagnosed with local or regional disease, but not distant disease.

## 1. Introduction

It is estimated that about 73,870 new cases of melanomas will be diagnosed in the United States in 2015 [1]. Approximately 9940 cases are expected to die from the disease. About 58% of the cases and 67% of the deaths involved males [1]. From 2000 through 2012, the estimated annual percent change in age-adjusted incidence rates of melanoma was 1.91 (*p* < 0.0001) in White males and 1.32 (*p* = 0.0008) in White females [2]. There was no significant change in rates for males or females in other racial groups. During these years, the estimated annual percent change in age-adjusted mortality rates of melanoma was 0.6 (*p* = 0.0023) for White males, but was not significant for White females, or males or females of other racial groups [3].

Following a diagnosis of melanoma, the physician can inform the patient of their prognosis, according to indicators such as age and tumor stage. After a patient has already lived some time beyond their initial diagnosis, their survival probability tends to improve. Survival estimates conditioned on already having survived some period of time allows those with cancer to update their prognosis and better determine how long after diagnosis it takes before they are considered cured. The current study presents conditional relative survival for melanoma, diagnosed during 2000–2008 and followed through 2012. Relative survival probabilities conditioned on having already survived one or more years (conditional survival) are reported, with the effect of selected demographic variables considered.

## 2. Results

**Figure 1:**
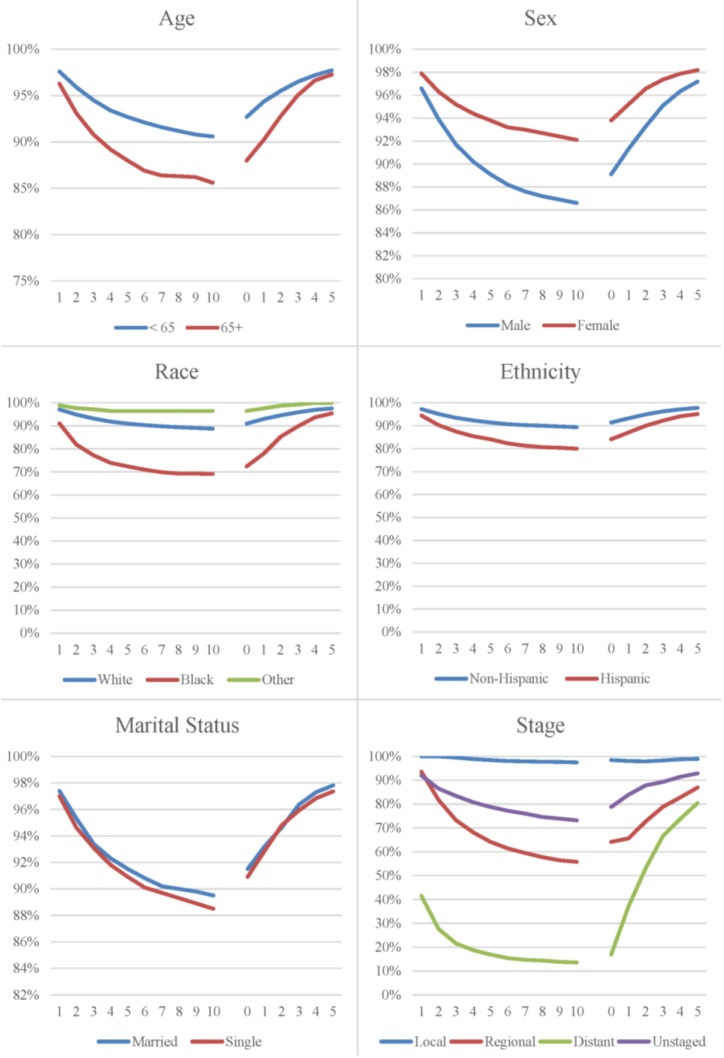
Relative (**left**) and conditional relative (**right**) survival curves for melanoma.

The distribution of melanoma cases are presented by age, sex, race, ethnicity, marital status, stage at diagnosis, and year of diagnosis (Table 1). Five- and ten-year relative survival decreases with age, stage at diagnosis, and is lower among males, Blacks, and Hispanics. Marital status at diagnosis and year of diagnosis had little influence on relative survival. Relative survival indicates the likelihood that cancer patients will not die from their cancer; it measures the survival of the patient cohort compared with the survival of the general population having the same characteristics with respect to age, sex, race, and calendar period. The relevance of relative survival (or observed survival) from a clinical perspective is limited. Once a patient has already survived some period of time, their prognosis changes. Relative survival and five-year relative survival conditional on having already survived one or more years after diagnosis are presented in Figure 1. Five-year conditional relative survival improves with each year already survived. The potential for improvement in five-year conditional relative survival is greatest for the level of variables with initially poorer survival, such as in older age, males, Blacks, Hispanics, and later staged cases. As five-year relative survival conditional on having already survived sometime after diagnosis increases toward 100%, it approaches the survival of the general population. For example, five-year relative survival is 72% at the time of diagnosis, but for patients who have already survived five years; their five-year relative survival is 95%.

**Table 1:** Melanoma cases diagnosed during 2000–2008 and relative survival through 2012 according to selected variables.

Variable				
	No.	%	5-Year Survival %	10-Year Survival %
Age				
<65 years	74,882	66.2	93	91
≥65 years	38,182	33.8	88	86
Sex				
Male	62,803	55.5	89	87
Female	50,261	44.5	94	92
Race				
White	107,198	94.8	91	89
Black	565	0.5	72	69
Other	5301	4.7	97	97
Ethnicity				
Hispanic	3571	3.2	84	80
Non-Hispanic	109,493	96.8	91	89
Marital Status				
Married	59,415	52.5	92	90
Single	53,649	47.5	91	89
Stage at Diagnosis				
Local	92,786	82.1	99	98
Regional	11,743	10.4	64	56
Distant	4206	3.7	17	14
Unknown	4329	3.8	79	73
Year of Diagnosis				
2000–2002	34,648	30.6	91	89
2003–2005	37,762	33.4	91	89
2006–2008	40,654	36.0	91	

Source: Surveillance, epidemiology, and end results program, 18 registries, 2000–2008.

Multiple regression analysis was used to assess the association between five-year relative survival conditioned on (0–5) years already survived and selected independent variables: years already survived, age, sex, race, ethnicity, marital status, and year of diagnosis. Year of diagnosis was not significant and, subsequently, dropped from the model. Significant interaction terms were observed between stage and age (*p* < 0.0001), sex (*p* < 0.0001), ethnicity (*p* = 0.0426), and marital status (*p* < 0.0001). Hence, regression models were stratified by stage at diagnosis (Table 2). In the unadjusted model, mean five-year conditional relative survival was not affected by years already survived for local disease (*p* = 0.4614), increased by 4.9% (*p* < 0.0001) per year already survived for regional disease, and increased by 13.1% (*p* < 0.0001) per year already survived for distant disease. In the adjusted model, mean five-year conditional relative survival showed a similar pattern. For local disease, five-year conditional relative survival was significantly lower in ages greater than 65 years (*p* = 0.0065) and in Blacks (*p* < 0.0001). It was significantly higher in females (*p* < 0.0001), non-Hispanics (*p* < 0.0001), and married individuals (*p* < 0.0001). Age had a greater inverse relationship with five-year survival in regional and distant staged disease (*p* < 0.0001). A similar result exists for females and for married individuals (*p* < 0.0001). On the other hand, non-Hispanics had better five year survival if diagnosed with local (*p* < 0.0001) or regional (*p* < 0.0001) disease, but not distant disease (*p* = 0.8929).

**Table 2:** Five-year relative survival conditioned on (0–5) Years Already Survived (YAS).

Tumor Stage Variable	Local (%)	Regional (%)	Distant (%)	Unstaged (%)
*Unadjusted Model*				
Intercept	**98.1**	**62.5**	**20.2**	**79.6**
YAS	0.0	**4.9**	**13.1**	**2.4**
*Adjusted Model*				
Intercept	**93.4**	**52.5**	**16.9**	**65.8**
YAS	−0.0	**4.7**	**12.8**	**2.3**
Age				
<65 years	0.0	0.0	0.0	0.0
≥65 years	**−0.7**	**−8.4**	**−8.1**	**−10.7**
Sex				
Male	0.0	0.0	0.0	0.0
Female	**1.2**	**6.9**	**4.1**	**6.1**
Race				
White	0.0	0.0	0.0	0.0
Black	**−9.1**	−7.6	−15.8	−11.8
Other	1.0	−2.6	10.0	**8.6**
Hispanic				
Yes	0.0	0.0	0.0	0.0
No	**4.0**	**7.9**	0.7	**15.4**
Marital Status				
Single	0.0	0.0	0.0	0.0
Married	**0.9**	**5.5**	**8.4**	−0.3

Source: Surveillance, epidemiology, and end results program, 18 registries research data, 2000–2008 diagnosed cases and followed through 2012. Estimates for each model were simultaneously calculated, adjusted for the other variables in the model. Bolded estimates are statistically significant, *p* < 0.05.

## 3. Discussion

This study presented conditional relative-survival rates for melanoma according to selected variables. The results may serve as a guide for patients and their physicians who are interested in an update of cancer prognosis as they survive some period of time after their initial diagnosis. As is the case for cancers of the prostate, corpus uteri, Hodgkin lymphoma, urinary bladder, breast, rectum, colon, five-year relative survival conditioned on already having survived five years exceeds 90%–95%, thus, nearing the survival of the general population [4]. As seen in other studies, the prognosis for melanoma survivors increased with each additional year of survival following diagnosis [4,5], except for those with local disease at diagnosis, who did not experience excess mortality during follow-up.

Stage of disease at diagnosis had the largest effect on conditional relative-survival rates, as consistent with previous research [4,5,6]. While there was no change in conditional relative survival for localized disease, there was a 4.7% change for regional disease and a 12.8% for distant disease.

The association between the demographic variables and conditional relative survival was dependent on stage at diagnosis. For example, older age had a more negative effect on conditional relative survival among patients who had regional or distant disease than in those with local disease; females compared with males had a better conditional survival, more so among regional and distant staged cases; Hispanics had worse five-year conditional relative survival, but only in local and regional staged patients; and married patients had better five-year conditional relative survival in later staged cases.

The poorer survival rates for Black *versus* White melanoma patients are consistent with previous research [7]. Because of small numbers of Blacks with melanoma, the poorer survival rates were only statistically significant for local staged disease. However, the lower survival rates were of practical importance in regional and distant staged cases. The current study also found that Hispanic melanoma patients have poorer prognosis than non-Hispanic patients, which is also consistent with previous research [8,9]. Poorer survival among Blacks and Hispanics is likely the result of delayed diagnosis and worse prognosis at the time of detection [9,10]. The current study adds to previous research by showing that while the poorer survival in Blacks is seen across the three broad stages of disease at diagnosis, the poorer survival in Hispanics is just in local and regionally staged patients.

Melanin is produced in cells in the skin called melanocytes. It is a primary determinant of skin color in humans with darker skin. Melanin pigment can be beneficial to the skin by protecting melanocytes and the outer structure of the skin against toxic chemicals, free radicals, and metal cations [11]. Although melanin pigment is protective against several types of insults to the melanocytes and skin, they make melanomas resistant to standard treatments (e.g., chemotherapy, radiotherapy, and phototherapy) because of the scavenging and radio-protective functions of melanin [12]. Thus, although melanin pigment is protective against melanoma skin cancer, it can hinder the effectiveness of various treatments for the disease, as consistent with the results of the current study.

Married compared with non-married melanoma patients have better survival. Previous studies support this finding. A study investigating the relationship between marital status and cancer survival found that unmarried patients, including those who are widowed, are at significantly greater risk of metastatic cancer, under treatment, and death resulting from their cancer than patients who are married [13]. A previous study of SEER data found that married women were less likely than married men to have a late stage diagnosis of melanoma, but there was no difference between unmarried men and women. Additionally, married women were less likely to die from melanoma than were married men, but there was no difference between unmarried men and unmarried women [14]. While these other studies established marriage as a protective factor for melanoma survival, the current study adds that the better survival is much more pronounced in regional and distant staged cases.

## 4. Methods

Analyses were based on 113,064 diagnosed cases of melanoma during 2000 through 2008, obtained from medical records at hospitals and other facilities by population-based cancer registries in the Surveillance, Epidemiology, and End Results (SEER) Program of the National Cancer Institute [15]. The SEER Program was established in response to the National Cancer Act of 1971 that mandated public health surveillance of cancer in the United States for use in prevention, diagnosis, and treatment of cancer. The cancer registries represented in this study are San Francisco, Connecticut, Detroit (Metropolitan) Hawaii, Iowa, New Mexico, Seattle (Puget Sound), Utah, Atlanta (Metropolitan), San Jose-Monterey, Los Angeles, Alaska Natives, Rural Georgia, California (excluding San Francisco, San Jose-Monterey, Los Angeles), Kentucky, Louisiana, New Jersey, and Greater Georgia. These population-based cancer registries cover approximately 28% of the United States population [16]. The SEER cancer registries routinely abstract records of all cancer patients in hospitals, clinics, nursing homes, and other health service units that provide diagnostic or treatment services; from private pathology laboratories and radiotherapy units; and from death certificates. Data collected by the tumor registries include patient demographics, tumor characteristics, extent of disease, and active patient follow-up of vital status including cause of death. Cancer coding is in accordance with the International Classification of Disease for Oncology Second Edition (ICD-O-2) [17].

SEER*Stat 8.2.1 software was used to calculate survival probabilities from any cause of death [18]. Relative survival probabilities were estimated. Relative survival is the observed survival adjusted for expected mortality from any cause [19]. Cases in the database were selected into the study if they were actively followed, had malignant behavior, were the first primary, and had known age. All death certificate only and autopsy only, and those alive with no survival time were excluded from the study. Patients diagnosed during the study period were followed through 2012.

Relative survival probability estimates were conditioned on having already survived one or more years (conditional survival). Five-year conditional relative survival estimates were obtained for patients each year from one year following initial diagnosis through five years following initial diagnosis.

Multiple regression analysis was used to assess the association between five-year relative survival conditioned on (0–5) years already survived and years already survived, age, sex, race, ethnicity, marital status, and year of diagnosis. Interaction terms were also assessed for statistical significance. Data were analyzed using the statistical software package PC-SAS (version 9.4; SAS Institute, Inc., Cary, NC, USA; 2014). The generalized linear models (GLM) procedure was used in SAS to perform the regression analysis. Model estimates were assessed using the t test and the 0.05 level of significance.

## 5. Conclusions

This study has provided data that supports previous research regarding melanoma survival. In addition, it showed the effect that selected variables have on conditional relative survival, according to tumor stage at diagnosis. This provides clinicians, and surviving patients help in evaluating individual chances for survival. There are studies available to show correlation between survival and the selected variables. However, this study is one of the first to show that these variables are still applicable in regards to conditional survival for melanoma patients.
